# Global Proteomics Deciphered Novel-Function of Osthole Against Pulmonary Arterial Hypertension

**DOI:** 10.1038/s41598-018-23775-8

**Published:** 2018-04-03

**Authors:** Li Yao, Yuxia Yang, Guanhong He, Chunqing Ou, Lan Wang, Kaixuan Liu

**Affiliations:** 1 Department of Medicinal Chemistry and Natural Medicine Chemistry, Department of Pharmacognosy, College of Pharmacy, Harbin Medical University, Harbin, 150081 China; 2 State-Province Key Laboratory of Biomedicine-Pharmaceutics of China, Harbin Medical University, Harbin, 150081 China

## Abstract

Pulmonary arterial hypertension (PAH) is a progressive cardiovascular-disease with high mortality lacking high-efficiency drug. Our efforts attempted to delineate therapeutic action of osthole produced by *Angelica Pubescens* Maxim, which has the capacity to treat PAH by exploiting an iTRAQ-based proteomic method. Excitingly, osthole was observed to significantly restore 98 of 315 differential proteins significantly modified by PAH progression. They were primarily annotated into 24 signaling pathways. Four mostly affected proteins (RPL15, Cathepsin S, Histone H3.3 and HMGB1) were experimentially validated which belonged to ribosome pathway, oxidative phosphorylation pathway, systemic lupus erythematosus pathway, complement and coagulation cascades pathway, whose modifications and modulations mostly accounted for therapeutic capacity of this compound against PAH. Altogether, our findings demonstrated that global proteomics is a promising systems-biology approach for deciphering therapeutic actions and associated mechanisms of natural products derived from traditional Chinese medicine. Importantly, osthole is supposed to be a candidate compound for new drug development to treat PAH.

## Introduction

Pulmonary arterial hypertension (PAH) is a complex multifactorial vascular disease with high mortality, which often yielded death to the patients at productive ages and causing tremendous financial cost^1–3^. According to the official report issued by World Health Organization (WHO), there are approximate 100-millions patients with PAH, the disease’s morbidity is much higher than that with patients infected with HIV or scleroderma^4^. The statistics showed that the 1-year mortality is 9–14% for idiopathic, heritable, and anorexigen-induced PAH, and about 10–30% for systemic-sclerosis-associated PAH, and similar mortality to metastatic breast cancer for severe PAH^5^.

Over the past twenty years, there are nine selected drugs that are observed to be efficient for treating PAH by targeting the known signaling pathways of endothelin, nitric oxide and prostacyclin, respectively. However, these drugs are mostly palliative as their therapies are often invalid for PAH. Therefore, new drugs are urgently needed for high-efficiency therapy against PAH progression^1^. PAH progression often involved the modifications in multiple targets and signaling pathways and pathogenesis, if the candidate compounds can simultaneously target multiple therapeutic pathways, they must significantly enhance therapeutic actions upon this condition.

**Figure 1:**
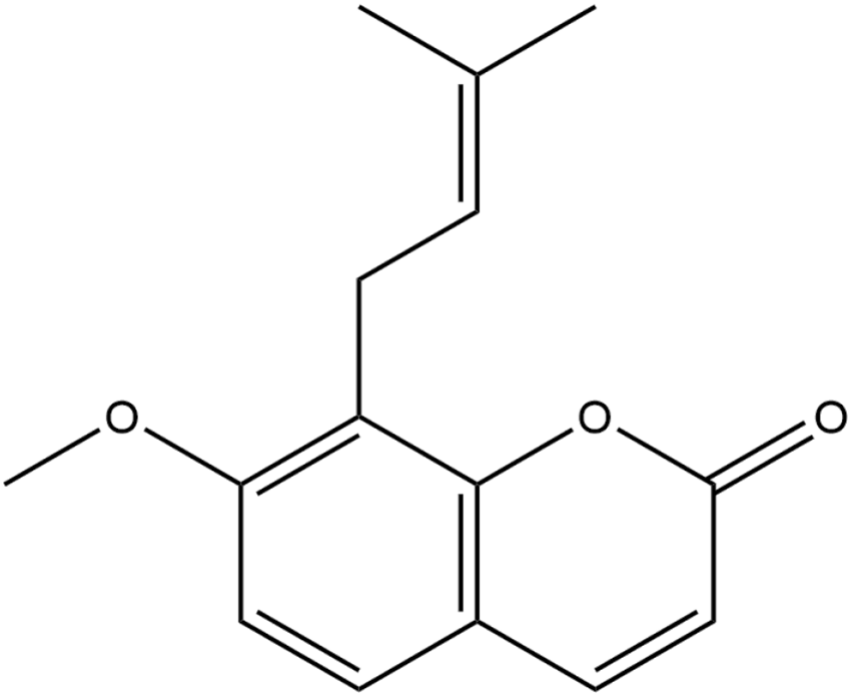
Chemical structure of osthole.

*Angelica pubescens* Maxim (APM) is a Chinese medicinal plant that has been broadly employed for eliminating wind, removing dampness, promoting blood circulation, and dispelling cold to relieve pain in clinical for hundred years. This medicine was also evidence with transiently hypotensive-effect^6^. Osthole is a mainly bioactive compound of APM (Fig. 1). There is a line of evidence to manifest that osthole can confer a diversity of pharmacological activities involving anti-inflammation, anti-virus, anti-tumor, anti-hepatitis and vasorelaxing activity^7–9^. However, whether osthole has therapeutic capacity against PAH, it remains unclear.

Currently, systems biology driven proteomics method is being emerged as a powerful and robust tool for recognizing and characterizing differential proteins associated with differently physiological and pathological events or processes, by which we can discover and identify protein biomarkers for disease diagnosis, as well as interrogate potentially therapeutic targets for drug discovery and development^10^. In addition, this method is also increasingly employed to investigate pharmacological actions and associated mechanisms of Chinese herbal medicines from proteome perspective^11^. Pulmonary pressure is an important factor in initiating signaling pathways leading to up-regulation of capZα, cathepsinD, annexin A4 and muscularization in the pulmonary vasculature in hypoxic induced PAH lung^12^. Moreover, There is growing evidence confirmed that BMPR2, C4a complement des Arg, C4 binding protein, C3 complement hydroxyl pyruvate, SULT1A1, NADH dehydrogenase, Fibrinogen A alpha, Fh1proteins and contractile machinery, and reactive oxygen species (ROS) whose production were correlated with the PAH progression^10,13–18^. Furthermore, many studies illustrated that PAH progression was closely associated with metabolic reprogramming and inflammatory process as well^19,20^.

In previous study, ethanol extract of APM was observed to exert hypotensive effect, however whether osthole is capable reducing pulmonary artery pressure, and associated mechanisms of action, are poorly understood. This study was designed to investigate therapeutic actions and associated mechanisms of osthole in rats with PAH induced by experimentally monocrotaline (MCT). Our preliminary data confirmed that osthole was able to reduce pulmonary arterial pressure, inhibit right ventricular hypertrophy and vascular remodeling. Global proteome assay further demonstrated that osthole was observed to significantly restore the modifications of Histone H3.3, High-mobility group protein B1 (HMGB1), Cathepsin S, and 60S ribosomal protein L15 (RPL15) during PAH progression. Those mostly affected proteins were covered by several key signaling pathways involving ribosome pathway, oxidative phosphorylation pathway, systemic lupus erythematosus pathway, complement and coagulation cascades pathway as this compound has the capacity to treat PAH by improving the modifications with those key pathways. Altogether, our findings manifested that global proteome method is supposed to be a promising approach for assessing systems actions of natural products and verified osthole could be a candidate compound for new drug discovery to treat PAH in clinic.

## Materials and Methods

### Chemicals and Reagents

Reference compound of osthole was purchased from National Institute for the Control of Pharmaceutical and Biological Products (NICPBP, Beijing, China); Monocrataline (MCT) was purchased from Sigma-Aldrich Co. (Missouri, USA). The following reagents and materials were used in this study. Urea (GibcoBRL); EDTA (Amesco); PMSF (Amesco); DTT (Promega); IAM (Promega); G250 (Amesco); Acrylamide (SIGMA); SDS (SIGMA); Methylene diacrylamide (SIGMA); TEMED (SIGMA); Bromophenol blue (SIGMA); iTRAQ® Reagent-8 Plex Multiplex Kit (Applied Biosystem); strata-X C18 (phenomenex); strong cation exchange (phenomenex Luna SCX 100 A).

### Ethics Statement

All the protocols were used for animal experiments, which were officially approved by the Ethical Committee of Laboratory Animals, Harbin Medical University (Harbin, China), and they were in compliance with Chinese National Regulations on the Use of Experimental Animals.

### Animals

6 weeks Wistar rats (male, weight at 200 ± 20 g) were purchased from Experimental Animal Center of Harbin Medical University, which is fully accredited by Institutional Animal Care and Use Committee (IACUC) of Harbin Medical University (Harbin, China) (License No.20001024). According to experimental design, we carried out the following animal experiments as there were three experimental groups with PAH group (n = 10) and treated group with osthole (n = 10) were first to receive a single injection of monocrotaline (MCT) (60 mg/kg, i.p.) subcutaneously to induce PAH occurrence, while control group (n = 10) received equivalent volume of saline injection. Then the rats in treated group were orally administrated with 80 mg/kg osthole (dissolved in 0.5% CMC-Na) daily for consecutive 28 days after MCT injection, as other control and PAH groups were treated with equivalent volume of 0.5% CMC-Na. Our efforts were made to minimize animal suffering and to reduce the number of animals used. All experiments were performed in accordance with the guidelines for animal-subject-based biological experiments issued by the Institutional Review Board of Harbin Medical University and the Animal Subjects Review Committee of Harbin Medical University.

### Echocardiography

Transthoracic echocardiography was performed on ultrasound machine (Vivid 7, GE Medical, USA). All the experimental rats were anesthetized using an intraperitoneal injection of pentobarbital. Haemodynamic data was analyzed by pulse-wave doppler. Mean Pulmonary arterial pressure (mPAP) and Pulmonary arterial systolic pressure (PASP) were calculated based on haemodynamic data. Heart tissues of rats were harvested for detection of right ventricular hypertrophy index (RVHI) which was calculated by the formula of RVHI = RV/(LV + S).

### Histomorphology and Immunohistochemistry

On 28^th^ day at MCT post-injection, all the experimental rats were deeply anesthetized with sodium pentobarbital (100 mg/kg, i.p.), then perfused transcardially with chilled PBS and decapitated. The entire lung was quickly removed under temperature controlling condition, further washed with PBS to remove plasma proteins, then immersed in 4% paraformaldehyde for overnight fixation. The tissues were dehydrated and embedded in paraffin wax according to standard protocol. The paraffin blocks were cut into 5 μm pieces for staining with hematoxylin and eosin (H&E), Masson and other analyses. For immunohistochemistry, the 5 μm paraffin-embedded tissue pieces were deparaffinized and rehydrated in graduated alcohol and then the sections were incubated with SMα -actin antibody at a concentration ratio as 1:50. Parallel controls were run with PBS only. After an overnight incubation, sections were washed three times with PBS and then subjected to the secondary antibodies (1:200) for the goat anti-mice IgG. Then, sections were visualized with 3, 3-diaminobenzidine (DAB) and counterstained using hematoxylin. Brown and yellow colors indicated positive stains. The positive staining areas of SMα-actin immunoreactivity in the vascular walls were viewed with an Eclipse 600 Nikon microscope and photographed with a digital camera. Morphometric analysis was analyzed with image software (Image Pro Plus).

### Sample Collection for Global Proteome Analysis

The lower half of right lung was isolated and frozen under liquid nitrogen. The specimens were stored at −80 °C until protein extraction. To increase coverage and reproducibility of proteomics assay to the proteins produced by the tissues, in this study, we combined equal amounts of 10 different specimens from the same treatment group together as one typical sample was used for global proteome assay.

### Protein Extraction, Digestion and iTRAQ Labeling

Protein digestion and iTRAQ labeling were performed according to iTRAQ kit protocol (Applied Biosystems). The extracted proteins were reduced with 10 mM DTT and alkylated with 55 mM IAM. They were then precipitated by cold acetone, stored at −20 °C for 3 h and concentrated by spun down at 20,000 g for 30 min. The precipitates were suspended in buffer- solution (50% TEAB, 0.1% SDS). Then 100 μg protein solutions were digested with 1 μg/μl trypsin solution at 37 °C overnight and labeled with iTRAQ tags. Control group (n = 10), PAH group (n = 10) and Osthole treated group (n = 10) were labeled with iTRAQ117, iTRAQ118, and iTRAQ119, respectively.

### iTRAQ 2D LC-MS/MS

The peptide mixture was re-suspended in buffer A (10 mM KH_2_PO_4_, 25% ACN, pH 3.0), and then fractionated and separated under high pH condition using an Aquity UPLC system (Waters Corporation) connected on a reverse phase column (C18 column, 2.1 mm × 150 mm, 3.5 um; Waters Corporation). The column was equilibrated with buffer A solution for 15 min. HPLC separation was performed with a gradient program with buffer-solution B (10 mM KH_2_PO_4_, 2 M KCl, 25% ACN, pH 3.0) from 0% to 5% in 45 min. The column flow rate was maintained at 1 ml/min and column temperature was set at room temperature. Fractions were collected and dried completely in a vacuum concentrator at last. Nano LC-MS/MS analysis was performed on a Q-Exactive Mass Spectrometer (Thermo Fisher Scientific) equipped with an UltiMate 3000 nanoHPLC system (Dionex). The desalted fractions were loaded onto a analytical column [C18, 75 um × 100 mm 5 um, Agela Technologies] and separated using a mobile phase containing buffer-solution A (0.1% formic acid in water) and buffer-solution B (0.1% formic acid in acetonitrile). A flow rate of 400 nl/min was used. The gradient separation was performed as keeping 5% B for 10 min, 5% to 30% B for 30 min, 30% to 60% B for 5 min, 60% to 80% B for 3 min, 80% B for 7 min, 80% to 5% B for 3 min, and 5% B for 7 min. A full mass scan was performed in data-dependent mode using a Q-Exactive Mass Spectrometer.

### Western Blotting Assay

Lung tissues were lysed on ice with RIPA (Beyotime) containing 1% protease inhibitor PMSF. Lysates were ultrasonicated for 1 min and centrifuged at 12000 rpm for 10 min at 4 °C. The protein concentration of each supernatant was determined with Bio-Rad protein assay kit (BCA, USA) with bovine serum albumin (BSA) as standard. Protein samples were separated by SDS-PAGE and transferred to nitrocellulose membranes (Millipore, USA) and then blocked with a Tris-buffered saline buffer (Tris 20 mM, NaCl 150 mM, pH7.6 Tween-20 0.1%) containing 5% skimmed milk. The blots were incubated with appropriate primary antibodies of RPL15 (1:600), HMGB1 (1:2000), Histone H3.3 (1:2000), Cathepsin S (1:400) at 4 °C overnight, followed by incubation with secondary antibody (goat anti-rabbit IgG or goat anti-mice IgG) for 1 h at room temperature. The immune reactivity was detected by ECL and exposed to X-ray film. Immunoblots were scanned with BIO-RAD Quality-One software.

### Data Analysis and Visualization

All MS/MS spectra from the samples were screened by PD (Proteome Discover 1.3, thermo), and then were searched using MASCOT software, version 2.3.0. Search results were exported into Uniprot 2014_Rattus database for protein qualification and quantification. Gene Ontology (GO) functional classifications (http://www.geneontology.org/) were used to unravel differentially expressed proteins. KEGG (Kyoto Encyclopedia of Genes and Genomes) was utilized to analyze canonical pathways involved for those differentially expressed proteins. Search Tool for the Retrieval of Interacting Genes/ Proteins (STRING) (http://www.string-db.org/) database of physical and functional interactions was used to evaluate the interactions among the differentially expressed proteins. Statistical analysis was performed using SPSS. Group data were presented as mean ± S.M. E and were compared using an unpaired t-test or a one-way ANOVA. Statistical difference was set at p value < 0.05 and significant difference was set at p value < 0.01.

## Results

### Osthole significantly lowered mean Pulmonary Arterial Pressure and markedly inhibited Right Ventricular hypertrophy

**Figure 2:**
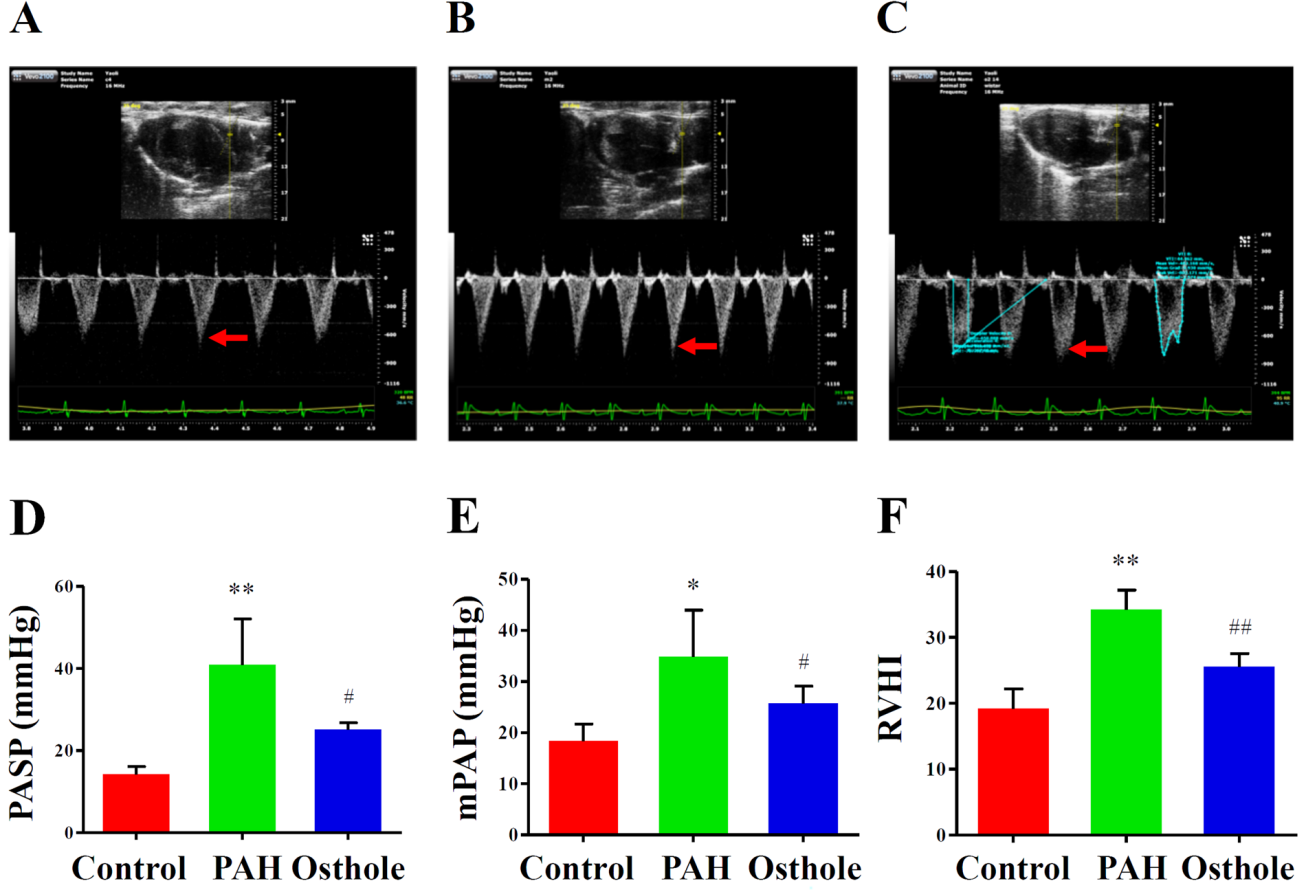
Osthole has the capacity to improve haemodynamics in rats with PAH. Representative Pulse-wave Doppler echocardiographs were selected from healthy-control rats (Control group) (**A**), the rats with PAH induced by MCT (PAH group) (**B**) and osthole treated rats with PAH (osthole group) (**C**). RVHI was calculated according to the formula of RVHI = RV/(LV + S). mPAP (**D**), PASP (**E**) and RVHI (**F**) were significantly increased in the rats with PAH compared with healthy rats, and such increase was markedly restored after osthole treatment. The data represented mean ± S.E.M. (p value < 0.05 was considered as statistically different between two groups (∗ or #), and p value < 0.01 (∗∗ or ##) was regarded as significantly different between two groups; ^*,**^was indicated to compare with control group; ^#,##^was designed to compare with PAH group).

PAH is defined as mean pulmonary artery pressure (mPAP) with a higher than 25 mmHg at rest and Pulmonary Arterial Systolic Pressure (PASP) is higher than 30 mmHg in human^21^. Our previous data revealed osthole to be a potential pulmonary vasorelaxant^8^. To further explore therapeutic capacity of osthole on PAH, we established a rat model with PAH induced by MCT injection. Pulse-wave Doppler Echocardiography was used to evaluate haemodynamic parameters and calculate mPAP and PASP^22^. To compare with the healthy controls, PASP was significantly increased to 40.88 mmHg in rats with PAH (PAH group) (p < 0.05) (Fig. 2B,D), but remarkably recovered to 23.15 mmHg by osthole treatment (p < 0.05) (Fig. 2C,D). Furthermore, mPAP was significantly enhanced to 34.84 mmHg in PAH group (p < 0.05) (Fig. 2B–E), but obviously restored to 25.72 mmHg by osthole treatment (p < 0.05) (Fig. 2C–E). RVHI in PAH group was significantly increased by 34.17% while compared with control group (Fig. 2B–F), but reversed to 25.56% by osthole treatment (p < 0.05) (Fig. 2C–F). In short, these results suggested that osthole greatly reversed the increased pulmonary arterial pressure and right ventricular hypertrophy caused by PAH progression.

### Osthole alleviated pulmonary vascular remodeling

**Figure 3:**
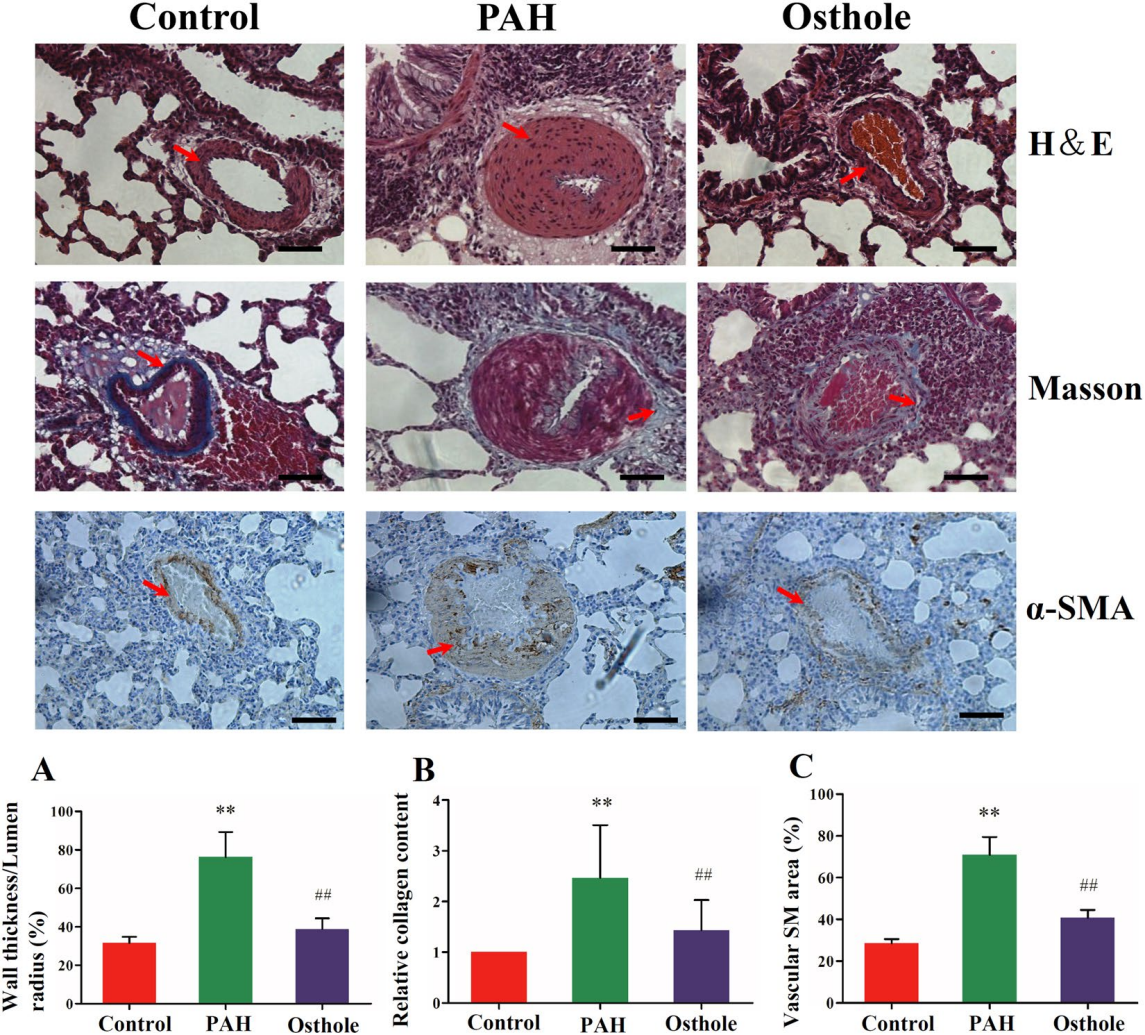
Systems influence of osthole on pulmonary vascular remodeling. (**A**) H& E staining of rat lungs demonstrated that wall thickness was increased in the rats with PAH compared with healthy-control rats. (**B**) Deposition of collagen was obviously higher in the rats with PAH than that in healthy ones which was characterized by Masson staining. (**C**) Expression of α-SM actin was also markedly increased in the rats with PAH. These differences between PAH and control groups were considerably restored by osthole treatment (80 mg/kg/d). Data shows quantitative analyses of positive staining per vascular area. The data was denoted as mean ± S.E.M. (p value <0.05 was considered as statistically different between two groups (∗ or #), and p value < 0.01 (∗∗ or ##) was regarded as significantly different between two groups; *^,^ ** was indicated to compare with control group; ^#, ##^ was designed to compare with PAH group).

Pulmonary vascular remodeling is characterized as a common pathological feature of PAH caused by a persisted increase in pulmonary artery pressure^23^. Histopathological changes were assessed by vascular wall thickness, deposition of collagen and appearance of cells expressing α-smooth muscle (α-SM) actin in partially muscularized or nonmuscularized small PAs. To compare with control group, pulmonary vascular wall thickness, especially the medial smooth muscle layer was apparently thicker in PAH group (HE staining) (Fig. 3A). Similarly, Masson staining showed that the deposition of collagen (blue) was massively increased in PAH rat small PAs (Fig. 3B). In addition, there was a significant increase in α smooth muscle actin staining (Fig. 3C), indicating the transformation of pulmonary arterial smooth muscle cells (PASMCs) from contractile phenotype to synthetic phenotype, which contributed to the proliferation of PASMCs and vascular muscularization of the medium. However, these increases including medial thickening and deposition of collagen were alleviated by osthole treatment, suggesting that osthole has the capability to inhibit pulmonary vascular remodeling.

### Global proteome assay revealed differential proteins underlying PAH progression and associated therapeutic potential of Osthole

**Figure 4:**
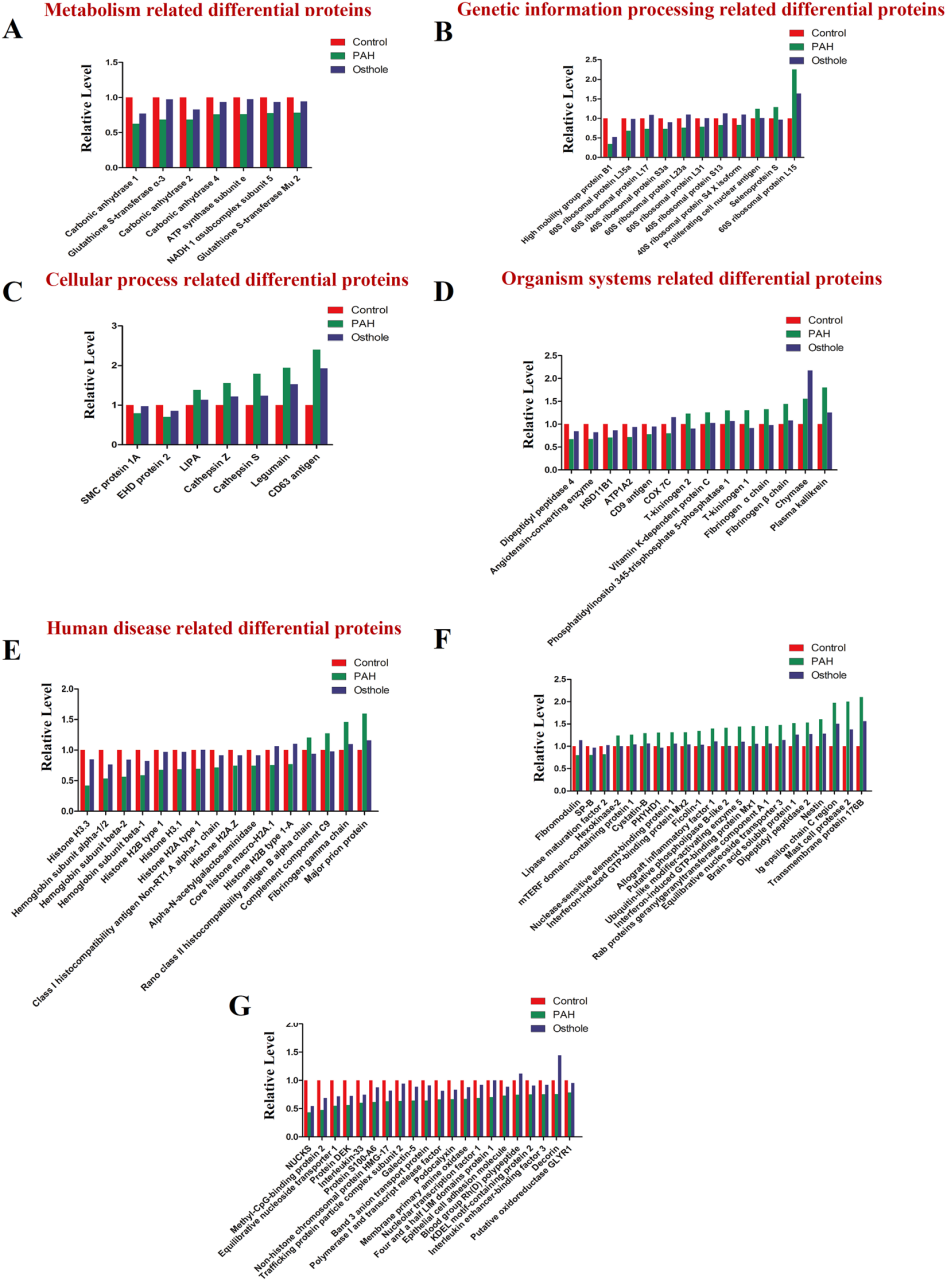
98 differential proteins modified by PAH progression that was significantly restored by osthole treatment. (**A**–**F**) Global proteome assay identified 98 differential proteins that were markedly modified PAH progression, while they were considerably restored by osthole treatment (fold of change >1.2 or <0.833, p value <0.05). (**A**) Metabolism covered differential proteins. (**B**) Genetic information related differential proteins. (**C**) Cellular progress associated differential proteins. (**D**) Organism systems related differential proteins. (**E**) Human diseases related differential proteins. (**F**,**G**) Many differential proteins were not covered by the known signaling pathways. Green bars represent differential proteins that were modified by PAH progression while compared to healthy controls; Blue bars indicate differential proteins modified by PAH, which were restored by osthole treatment.

To discover and identify differential proteins implicated in the progression of PAH, global proteome method was developed based on using iTRAQ labeling coupled with 2D-LC MS/MS, this method was used to discover and characterize 315 differential proteins (P < 0.05) whose level changes were able to significantly distinguish PAH from control groups. We found that 137 differential proteins were up-regulated in PAH (Fig. 4), including Myotubularin (Q6AXQ4, 2.79-fold), 60S ribosomal protein L15 (P61314, 2.25-fold) (Fig. 4B), transmembrane protein 176B (Q925D4, 2.102-fold) (Fig. 4F), etc. Moreover, 178 differential proteins were down-regulated in PAH (Fig. 4), such as High mobility group protein B1 (P63159, 0.343-fold) (Fig. 4B), and Histone H3.3 (P84245, 0.418-fold) (Fig. 4E), etc.

Furthermore, osthole treatment was observed to significantly restore 98 differential proteins to the levels of healthy controls while compared to PAH group (Fig. 4). Among 98 differential proteins, 38 differential proteins were up-regulated in PAH group but down-regulated caused by Osthole treatment, and 60 differential proteins down-regulated in PAH rats but up-regulated after Osthole treatment. We randomly selected Histone H3.3, HMGB1, RPL15 and Cathepsin S (Fig. 4C) for further validation using Western blotting assay.

### Expressional validation of selectively differential proteins

**Figure 5:**
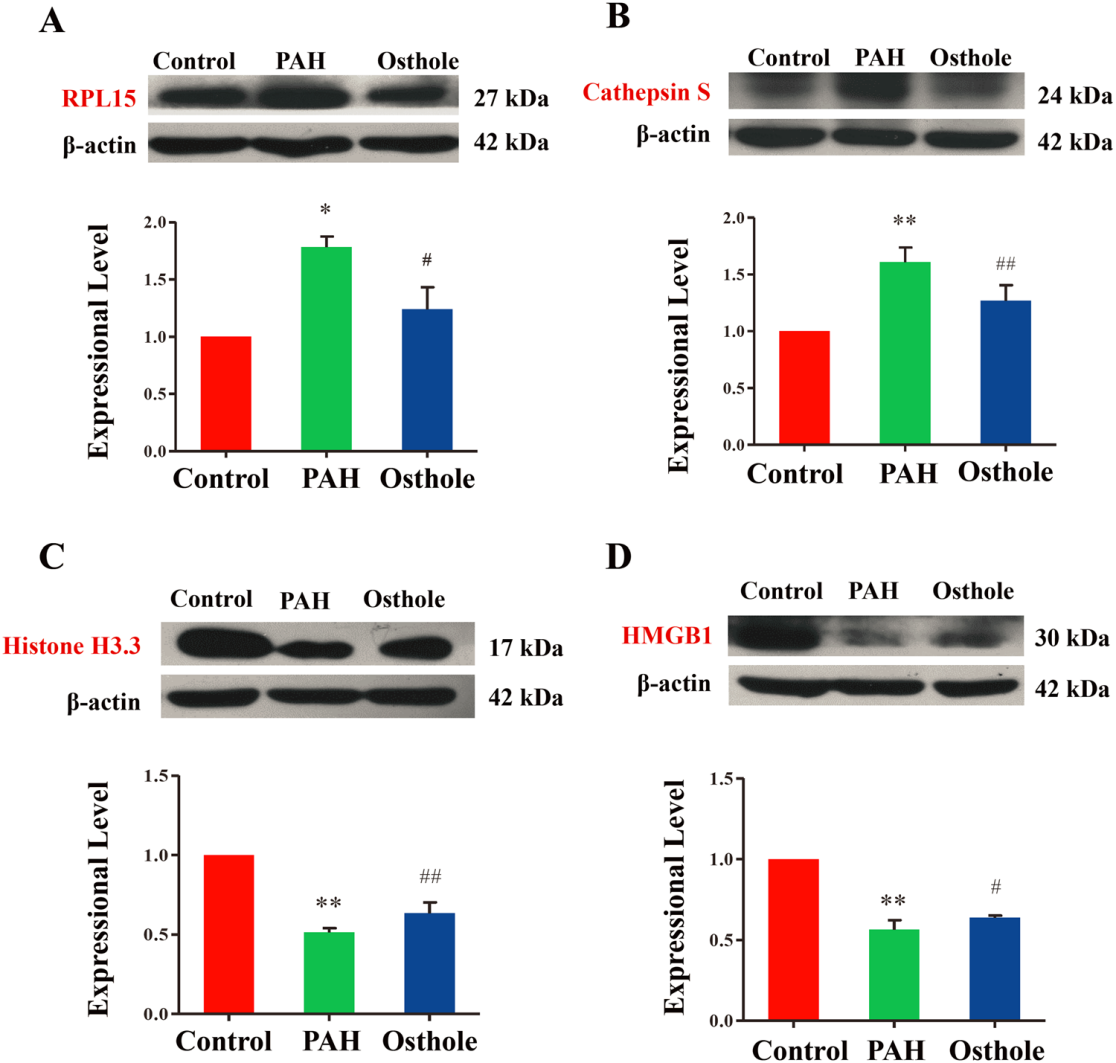
Experimental validation of typically differential proteins. (**A**) 60S ribosomal protein L15 (RPL15) expression in rats’ lungs. (**B**) Cathepsin S expression was expressed in rats’ lungs. (**C**) Histone H3.3 expression in rats’ lungs. (**D**) High mobility group box 1(HMGB1) expression in rats’ lungs. (p value <0.05 was considered as statistically different between two groups (∗ or #), and p value <0.01 (∗∗ or ^##^) was regarded as significantly different between two groups; ^*,**^was indicated to compare with control group; ^#,##^was designed to compare with PAH group).

Our results revealed that the expressional levels of RPL15 and Cathepsin S (Fig. 5A,B) were significantly increased when compared with the healthy controls, whereas Histone H3.3 and HMGB1 (Fig. 5C,D) were remarkably decreased while compared with healthy controls. The expressional levels of these proteins were significantly reversed by osthole treatment, which were completely consistent with the discoveries in iTRAQ-2D -LC-MS/MS based proteome assay.

### GO analysis of differential proteins responding to PAH progression and osthole treatment

The expressional profiles of differential proteins were analyzed with the tool of gene ontology (GO) annotation, which classified these differential proteins into three major categories: biological process, cellular component, and molecular function. Based on the analysis of biological processes, we found that differential proteins in PAH group were involved in single-multicellular organism processes and multicellular organismal processes (Figure S4). Analysis of cellular components manifested that 104 proteins supported membrane construction and 90 proteins were linked to cell periphery (Figure S2). These differential proteins in PAH group expressed a diversity of importantly molecular functions, such as 34 proteins for transition metal ion binding, 30 proteins for DNA binding, 28 proteins for structural molecule activity and 23 proteins for peptidase activities (Figure S3).

Furthermore, biological processes analysis in osthole treated group identified 74 proteins accounted for nitrogen compound metabolic processes and 70 proteins for single-multicellular organism processes (Figure S1). Additionally, cellular components analysis predicted that 66 proteins were involved in non-membrane-bound organelles and 66 proteins were hosted by intracellular non-membrane-bound organelles (Figure S5). In addition, we found that the differential proteins modulated by osthole treatment were involved in nucleic acid binding (40 proteins, 19.6%) and DNA binding (27 proteins, 31.2%) (Figure S6).

### Annotation of signaling pathways of differential proteins were modified by PAH progression and relevantly modulated by osthole treatment

**Figure 6:**
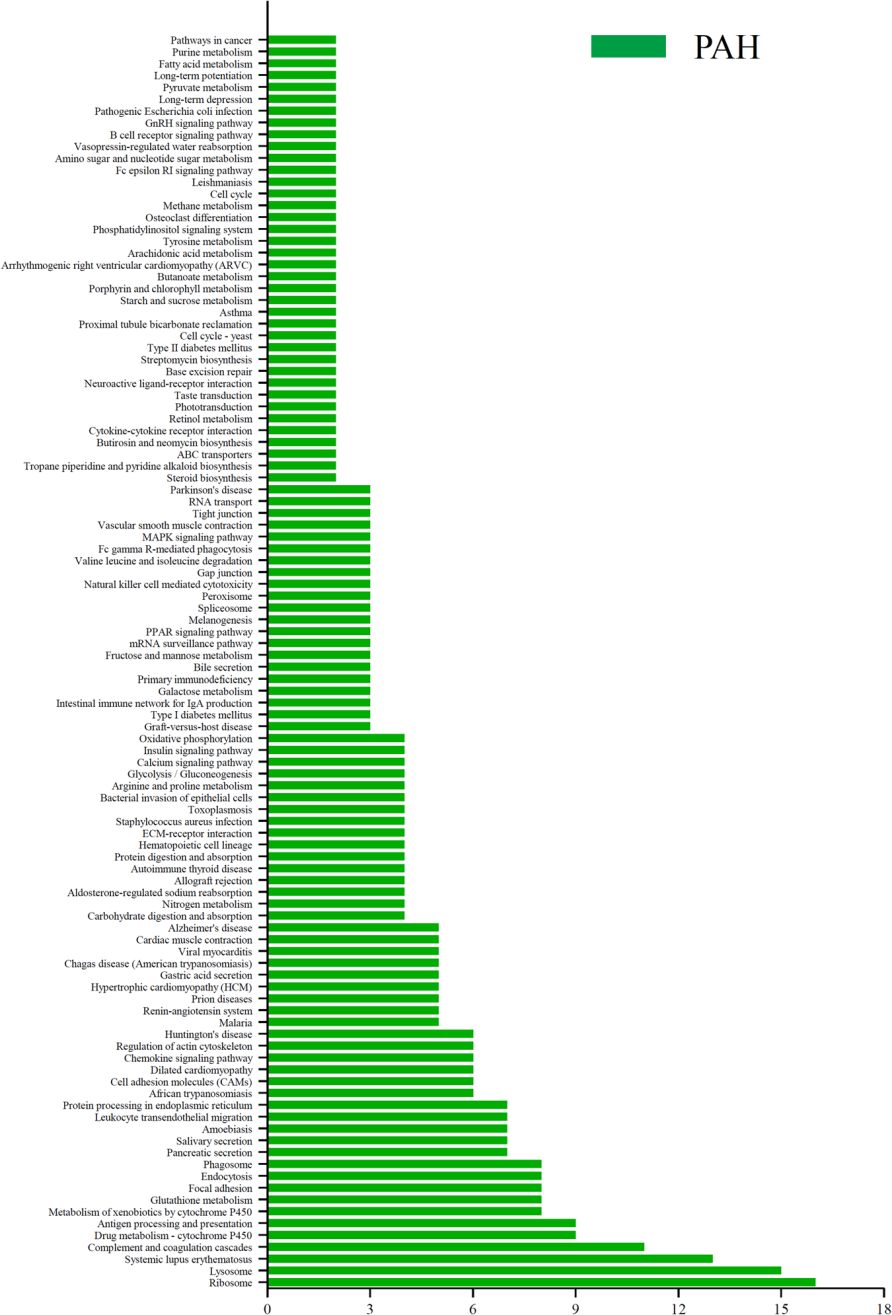
KEGG pathway analysis of differential proteins modulated by PAH development. Total 315 differential proteins were modified by PAH progression while compared to healthy controls, which were covered by106 signaling pathways. X axis is number of differential proteins hosted by targeted signaling pathways.

**Figure 7:**
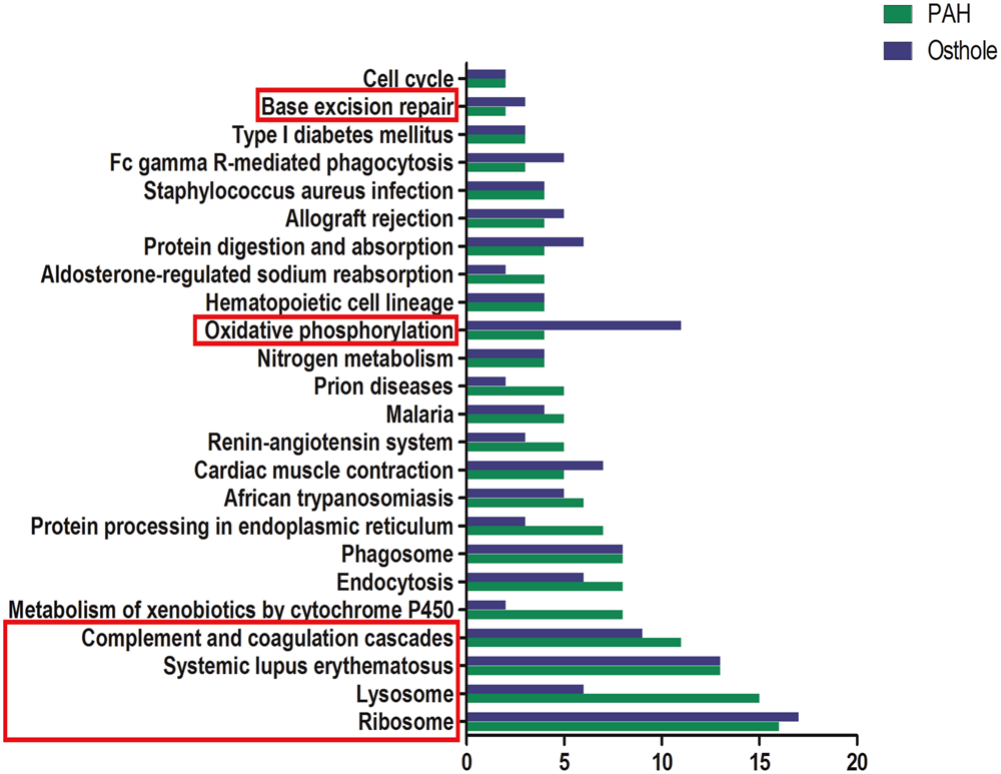
KEGG based signaling pathway analysis of differential proteins modified by PAH development, which were significantly restored by osthole treatment. 98 differential proteins were annotated into twenty-four signaling. The most affected signaling pathways were ribosome pathway, complement and coagulation cascades pathway, systemic lupus erythematosus pathway, lysosome pathway, oxidative phosphorylation pathway and base excision repair pathway highlighted in red bracket. X axis is for the number of differential proteins covered by the targeted signaling pathways. Green bars are the differential proteins modified by PAH progression. Blue bars are differential proteins that were restored by osthole treatment while compared to PAH group.

**Figure 8:**
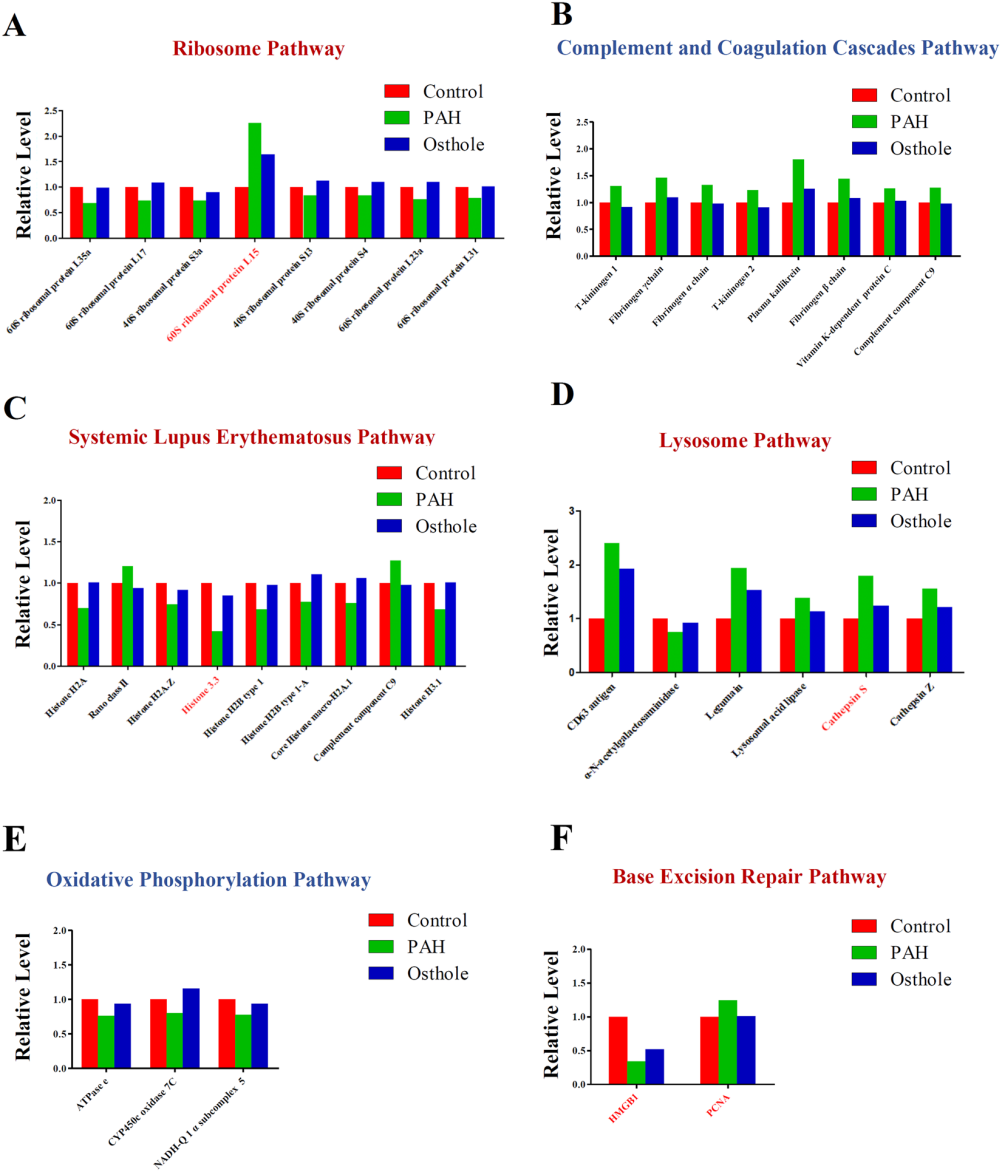
Six signaling pathways mostly accounted for the treatment of osthole upon PAH. (**A**) Ribosome pathway. (**B**) Complement and coagulation cascades pathway. (**C**) Systemic lupus erythematosus pathway. (**D**) Lysosome pathway. (**E**) Oxidative phosphorylation pathway and (**F**) base excision repair pathway in PAH were both listed.

To identify possible signaling pathways and biological processes involved the above differential proteins, we annotated the data with Kyoto Encyclopedia of Genes and Genomes (KEGG) as we discovered the signaling pathways mostly covered Metabolism, Genetic information processing, Environmental information processing, Cellular Processes, Organismal systems, Human diseases. Total 106 signaling pathways were significantly modified by PAH progression (Fig. 6) and 24 signaling pathways were modulated by osthole treatment (Fig. 7). Furthermore, 106 signaling pathways mostly accounted for PAH progression, as 23 signaling pathways covered by systems metabolism mainly involving glycolysis, lipid metabolism, amino acid and energy metabolism, 15 signaling pathways targeted immune responses, and 8 signaling pathways participated in many infectious processes. Our findings were almost agreeable with the previous publications as metabolic reprogramming and inflammation mostly accounted for PAH pathogenesis^20^. Ribosome pathway (16 proteins) (Fig. 8A), complement and coagulation cascades (11 proteins) (Fig. 8B), systemic lupus erythematosus (13 proteins) (Fig. 8C), lysosome (15 proteins) (Fig. 8D) were mostly affect signaling pathways by PAH progression. Total 24 signaling pathways were recoverably modulated by osthole treatment relevant to PAH progression, 3 signaling pathways were assigned to systems metabolism, such as oxidative phosphorylation (energy metabolism), nitrogen metabolism (energy metabolism), metabolism of xenobiotics by cytochrome P450 (xenobiotics biodegradation and metabolism); 3 signaling pathways were associated with genetic information processing: ribosome (transcription), base excision repair (replication and repair) (Fig. 8F), protein processing in endoplasmic reticulum (folding, sorting and degradation); 4 signaling pathways were correlated to with cellular processes: cell cycle (transport and catabolism), endocytosis (transport and catabolism), lysosome (transport and catabolism), phagosome (transport and catabolism); 7 signaling pathways were linked to with organismal systems: complement and coagulation cascades (immune system), Fc gamma R-mediated phagocytosis (immune system), Hematopoietic cell lineage (immune system), aldosterone-regulated sodium reabsorption (excretory system), cardiac muscle contraction (circulatory system), protein digestion and absorption (digestive system), renin-angiotensin system (endocrine system); and 7 signaling pathways were related to human diseases as systemic lupus erythematosus (infectious disease), African trypanosomiasis (infectious disease), malaria (infectious disease), staphylococcus aureus infection (infectious disease), allograft rejection (immune disease), prion diseases (neurodegenerative disease), type I diabetes mellitus (endocrine and metabolic disease). All in all, these results suggested that osthole has the capacity to ameliorate pulmonary arterial pressure and vascular remodeling by mostly promoting oxidative phosphorylation and nitrogen metabolism, remodeling metabolic reprogramming and inhibiting inflammation and immune responses. And ribosome (17 proteins) (Fig. 8A), complement and coagulation cascades (9 proteins) (Fig. 8B), systemic lupus erythematosus (13 proteins) (Fig. 8C), oxidative phosphorylation (11 proteins) (Fig. 8E) were mostly affected signaling pathways by osthole treatment, suggesting those pathways novel targets for better understanding the pathogenesis of PAH and therapeutic mechanisms of this promising natural product.

### Biological networking of differential proteins involved in PAH progression and osthole treatment

**Figure 9:**
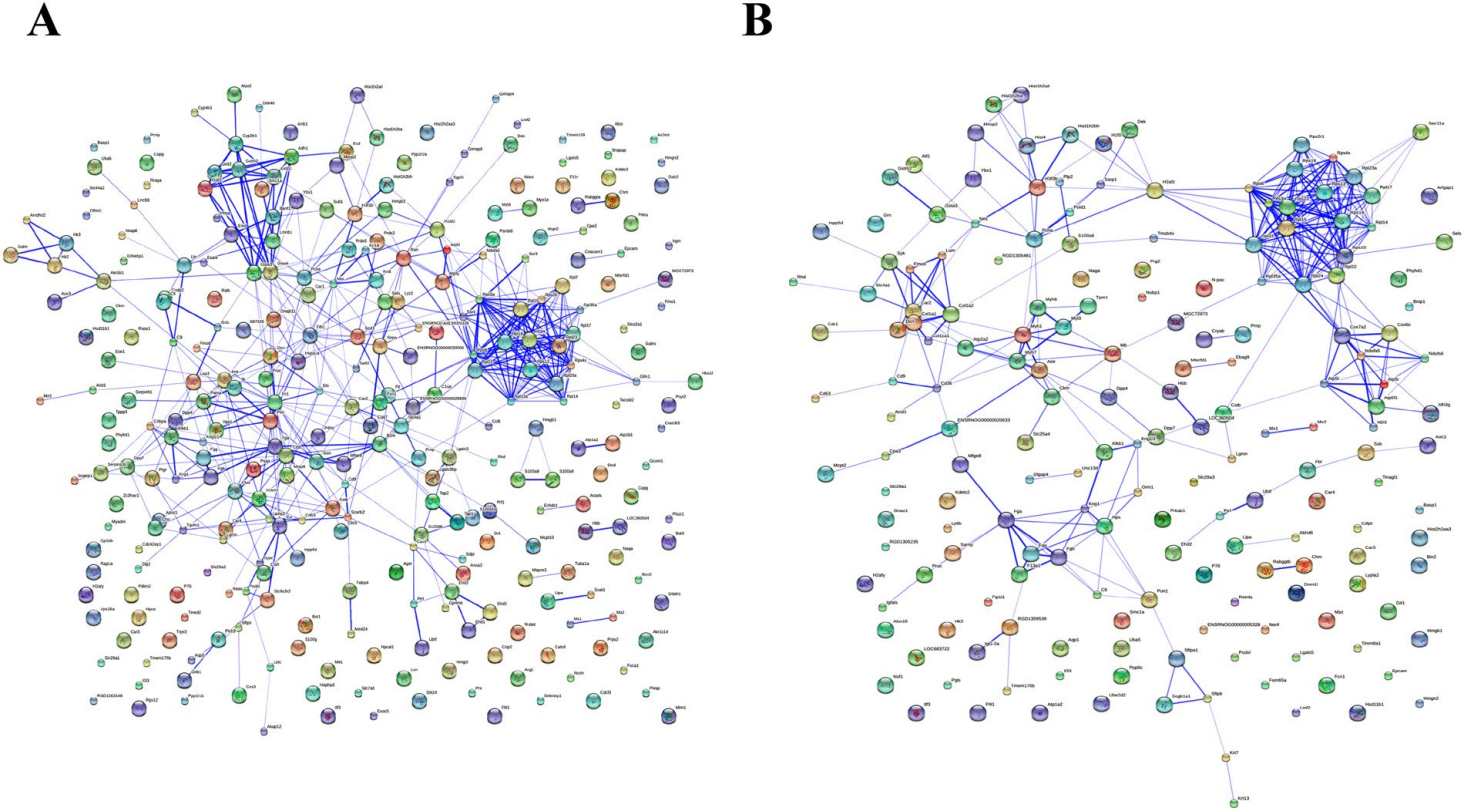
The biological networking of differential proteins was visualized with STRING. The interactions amongst differential proteins were implicated in PAH progression (**A**) and Osthole treatment (**B**). The networks are displayed graphically as nodes (differential proteins) and edges (biological relationships between the proteins).

A STRING network analysis was performed to interrogate the relationships among differential proteins. Within the resulted protein-protein network, several complexes and cellular functions together formed prominent and tightly connected clusters that were assessed via complex detection. The networks were visualized as nodes (proteins) and edges (the biological relationship between the proteins) in (Fig. 9), which phenotypically characterized the protein-protein interactions of PAH progression and osthole treatment, respectively. At last, our data revealed that 295 proteins were involved in functional and physical connections resulted from 315 differential proteins modified by PAH progression (Fig. 9A) and 46 proteins from 98 differential proteins modulated by Osthole treatment (Fig. 9B).

## Discussion

PAH is a complex condition that was characterized by pulmonary arteries remodeling, a further elevation in mPAP and consequently right heart failure and death^21^.

Corresponding treatment is facing the huge challenge due to it was extreme lack of high-efficiency drugs in clinic. We previous work preliminarily verified that APM has a great potential for anti-hypertension against PAH, however, bioactive compounds of APM and associated therapeutic mechanisms are still unclear^6,8^. Osthole, 7-methoxy-8-(3-methyl-2-butenyl) coumarin, one of bioactive compounds of APM, which rendered a broad of bioactivities as anti-cancer, anti-arrhythmia, anti-hepatitis and vasorelaxation activities^9^. Our previous study has confirmed osthole was a new pulmonary vasodilator in isolated rats and human pulmonary arterial rings, as well as its cardiovascular protective activity has been characterized through selectively inhibiting vascular smooth muscle cells proliferation^9^. Whereas we deduced that this compound might have the potential to treat PAH, but this required further verification by functional experiments. In this study, we were first to verified osthole was able to decrease pulmonary artery pressure, ameliorate right ventricular hypertrophy and inhibit vascular remodeling *in vivo*. Furthermore, we employed iTRAQ labeling combined with 2D LC-MS/MS facilitated proteomics method for investigating the pathogenesis of PAH and therapeutic discovery of osthole from differential proteins perspective. Our data revealed that 315 differential proteins were modified by PAH progression and of 98 differential proteins were restored to healthy controls by osthole treatment while compared to PAH group. Four proteins as Histone H3.3, HMGB1, RPL15 and Cathepsin S were significantly regulated by osthole treatment, those proteins might have the capacity for better understanding of the pathogenesis of PAH and therapeutic discovery of osthole. Furthermore, biological annotation of differential proteins revealed that 24 signaling pathways involving systems metabolism, immune response and infectious processes within 106 modified signaling pathways during PAH progression, such discovery was completely agreeable with previous publication as PAH progression incurred substantially metabolic reprogramming and inflammatory processes. We also found that osthole mainly restored the differential proteins to treat PAH, which mostly enriched in ribosome pathway, oxidative phosphorylation pathway, systemic Lupus erythematosus pathway, complement and coagulation cascades pathway as this compound was capable of promoting oxidative phosphorylation and modulating metabolic reprogramming, relieving oxidative stress and inhibiting inflammatory processes, which provided a new strategy to alleviate the vascular remodeling occurring in PAH progression.

It was observed that ribosome pathway is mostly affected signaling pathway covered numerous proteins that were closely associated with PAH progression and osthole treatment. Ribosomal proteins (RPs), a family of RNA-binding proteins, composed by two subunits: 40S small subunit with decoding function and 60S large subunit with catalyzing the formation of peptide bonds, which play key roles in regulation of protein synthesis, cell proliferation, apoptosis, DNA repair and other cellular processes^24–26^. Our data revealed that RPL15 was significantly up-regulated in PAH progression, whereas, RPL23a, RPL35a, RPL17, RPL31, RPS13, RPS4, RPS3a are noticeably decreased in PAH rats, and such expressional were significantly restored by osthole treatment, as RPL15 were reported to promote cell proliferation, which is a prognostic biomarker in pancreatic ductal adenocarcinoma^24^. Our data confirmed that RPL15 was remarkably enhanced in PAH progression, and markedly recovered by osthole treatment, suggesting osthole treated PAH by mostly modulating the expression of RPL15 to promote muscle cell proliferation and vascular remodeling.

PAH is increasingly confirmed as a complication of systemic lupus erythematosus (SLE). The association between PAH and SLE suggested that inflammatory process activate a proliferative and inflammatory pulmonary lesions, which eventually lead to vascular remodeling^20,27^. Activation of the complement system can directly contribute to vascular leakage, which was observed to be underlying PAH pathogenesis^12,28^. Our proteome data illustrated that plasma kallikrein (complement and coagulation cascades Pathway), complement component C9 and ranoclass II (both in systemic lupus erythematosus pathway), CD63 antigen, legumain, Cathepsin S, Cathepsin Z, lysosomal acid lipase(proteins in lysosome pathway) were highly enhanced in PAH, Whereas Histone H3.3, Histone H 3.1(proteins in systemic lupus erythematosus pathway), HMGB1 (base excision repair pathway) were considerably down-regulated in PAH progression, these proteins were markedly recovered by osthole treatment. In addition, we found that Cathepsin S is a cysteine proteinase that was implicated in the diverse tissue remodeling^29^. HMGB1, a DNA-binding protein, is ought to trigger inflammatory responses and promote tissue regeneration in rats when it was released into the extracellular space as it’s circulating level was significantly increased during the early stage of MCT or hypoxia-induced PAH^30–33^. Increased circulating HMGB1 and histones could play a significant role in contributing to systemic inflammation and multiple organ injury during severe acute pancreatitis^34^. Our proteomic assay demonstrated that Cathepsin S was overexpressed in rats’ lungs with PAH, but Histone 3.3 and HMGB1were markedly decreased accordingly, suggesting again activation of inflammation/immune responses were one of importantly development mechanisms of PAH^35^. Basically, immunomodulation and anti-inflammatory activity of osthole has been confirmed in late 1990 s^9^. Cardiovascular protection of osthole was also verified as it can exert vasorelaxant effect, antifibrotic effect and inhibits abnormal vascular cell proliferation^36^. In this study, osthole was visualized to have the capability to restore the expression of Cathepsin S, HMGB1 and Histone H3.3 with PAH progression, thus this compound was able to treat PAH by partially improving the inflammatory process, which was characterized by down regulation of Cathepsin S and up-regulation of Histone H3.3 and HMGB1 in PAH progression.

## Conclusion

PAH progression incurred substantially differential expressions of proteins, this might provide a new window for better understanding of PAH pathogenesis and associated therapeutic discovery. Our study was first to perform proteome assay to PAH progression and treatment of osthole in experimental setting. As our expectation, we found numerous differential proteins were implicated in PAH progression, the modifications in their signaling pathways are capable of deciphering the development mechanisms of this condition, they were also partially verified as novel targets for interrogating therapeutic actions of osthole. Therefore, we were capable of elucidating the development mechanisms of PAH and therapeutic capacity of osthole from differential proteome perspective. Altogether, our findings will provide better understanding of PAH pathogenesis and decipher therapeutic capacity of osthole.

## Electronic supplementary material

Supporting Data
