# Supplementary material for: Global Proteomics Deciphered Novel-Function of Osthole Against Pulmonary Arterial Hypertension
Source: Sci Rep. 2018 Apr 3;8:5556. doi: 10.1038/s41598-018-23775-8 (PMC5882969; doi:10.1038/s41598-018-23775-8)
Supplement: Supplementary file 1 — Supporting Data [file 41598_2018_23775_MOESM1_ESM.doc]

**Supporting Data:** Figures S1-S7

**Global Proteomics Deciphered Novel-Function of Osthole Against Pulmonary Arterial Hypertension**

Li Yao ^1^, ^2^*, Yuxia Yang ^1, #^, Guanhong He ^1, #^ , ChunqingOu^1^, Lan Wang ^1^, Kaixuan Liu^1^

^1^ Department of Medicinal Chemistry and Natural Medicine Chemistry, Department of Pharmacognosy, College of Pharmacy, Harbin Medical University, Harbin, 150081, China

^2^ State-Province Key Laboratory of Biomedicine-Pharmaceutics of China, Harbin Medical University, Harbin, 150081, China

^#^ The authors are equally contributed to this work.

***Corresponding author.**

Prof. Li Yao, College of Pharmacy, Harbin Medical University, 157 Baojian Road, Harbin, Heilongjiang 150081, China.

Tel: 86-186-86845106

E-mail: liyao.prof@hotmail.com

**Supplementary Figure Legends**

**Figures S1-S6. Gene ontology classification of differential proteins mostly accounted for the treatment of Osthole upon.** The biological classification of differential proteins was manipulated according to biological process (Figure S1 and S4), cellular components (Figure S2 and S5) and molecular function (Figure S3 and Fig S6).

**Figure S7. The full-length gels for western blots illustrated in Figure 5 A-D.**


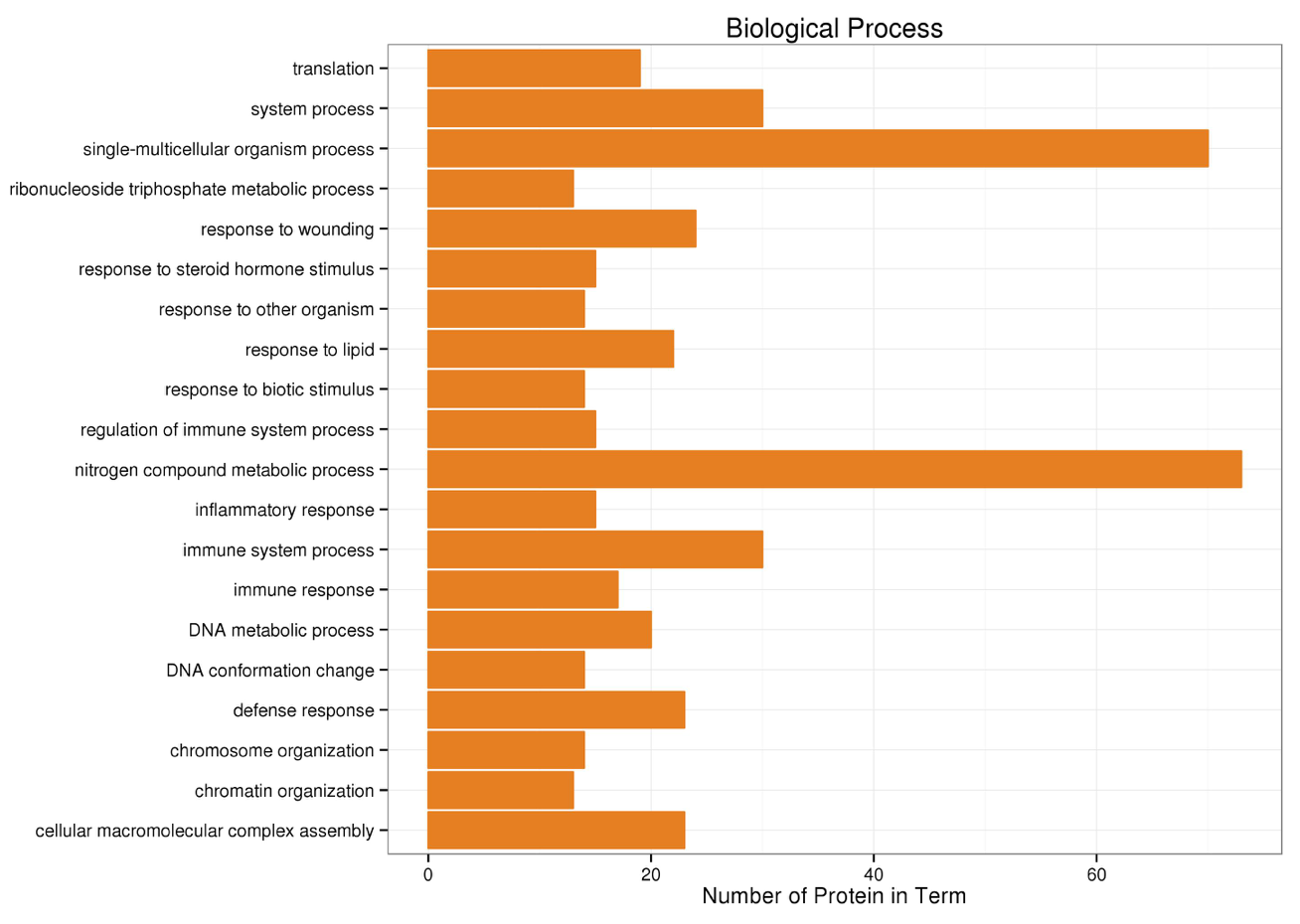


Figure S1


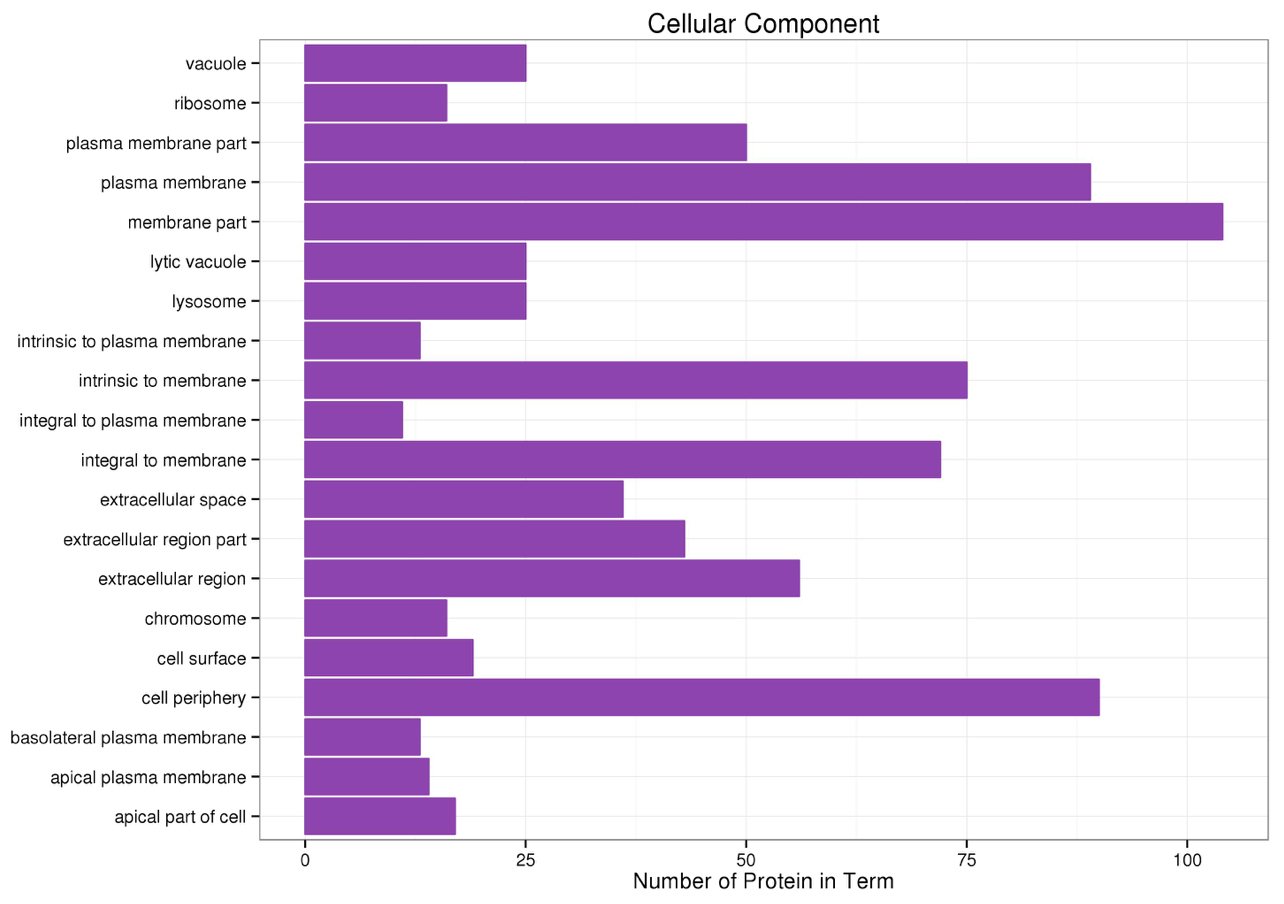


Figure S2


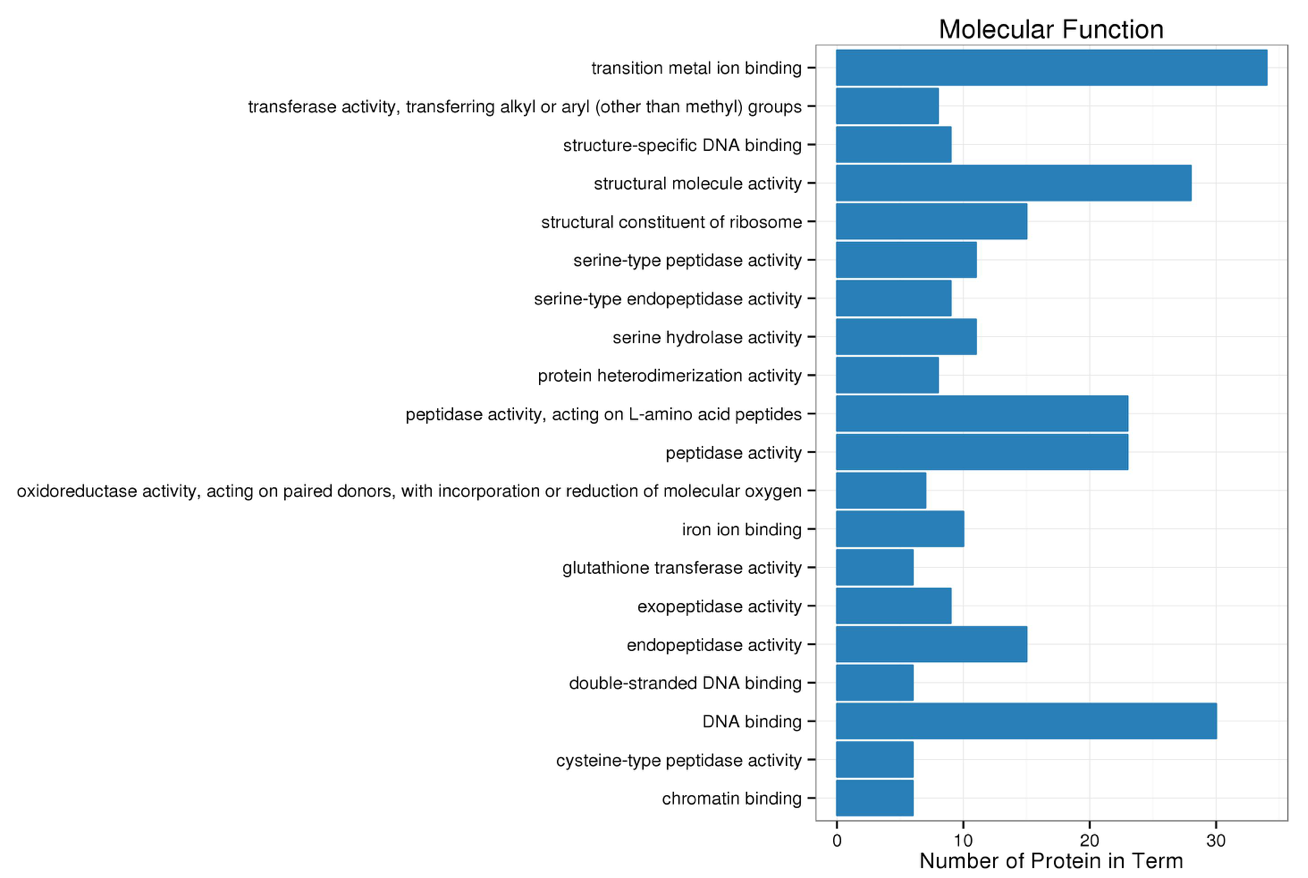


Figure S3


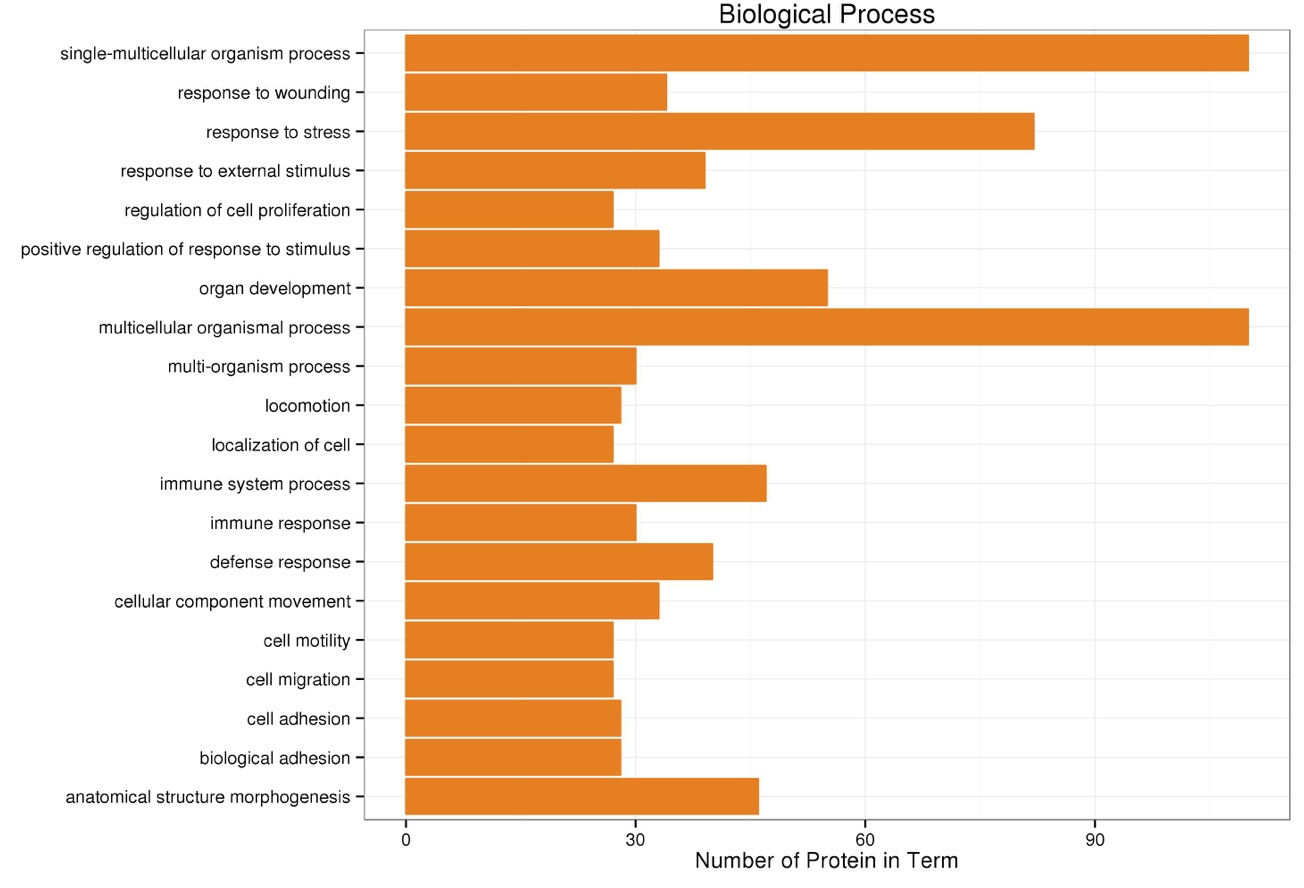


Figure S4


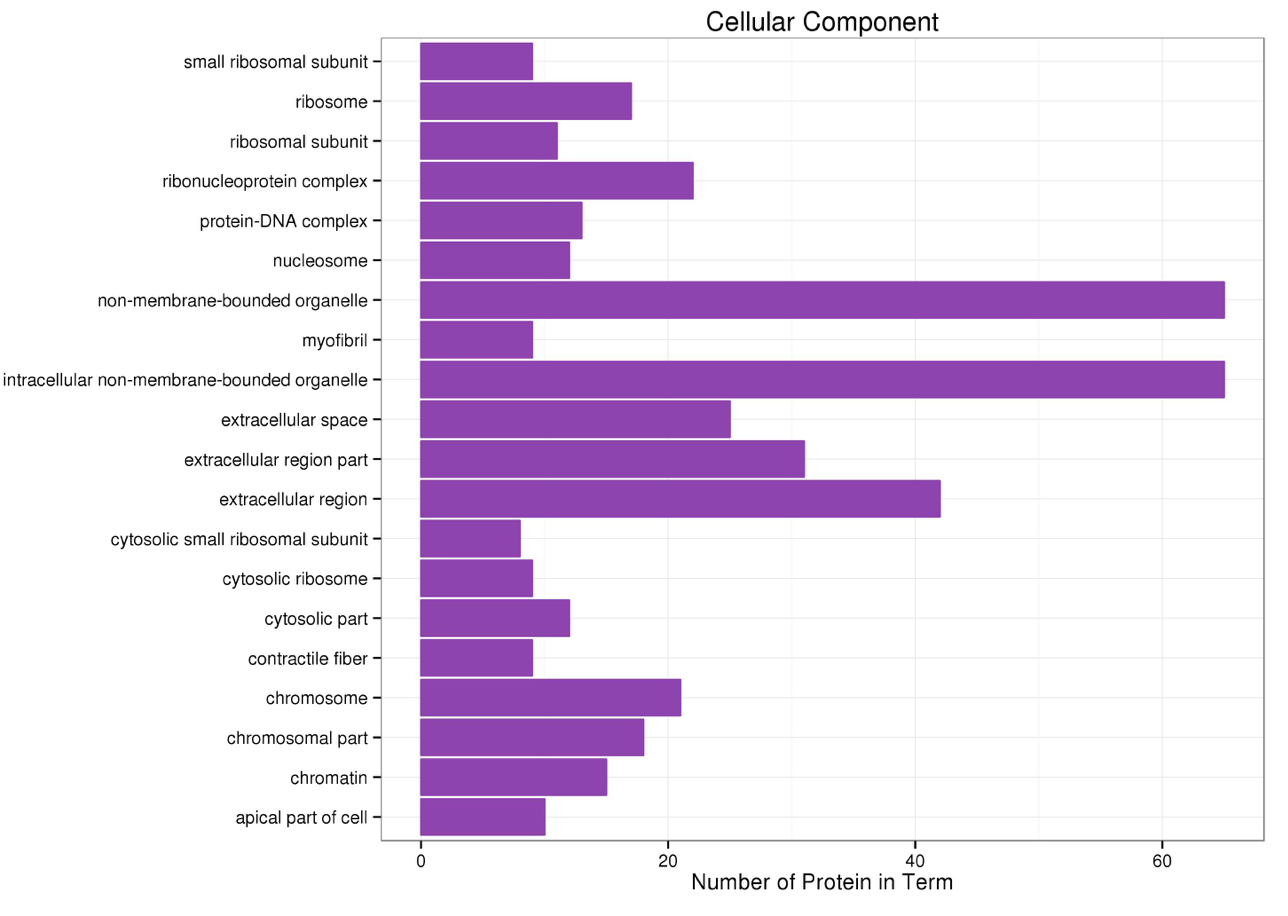


Figure S5


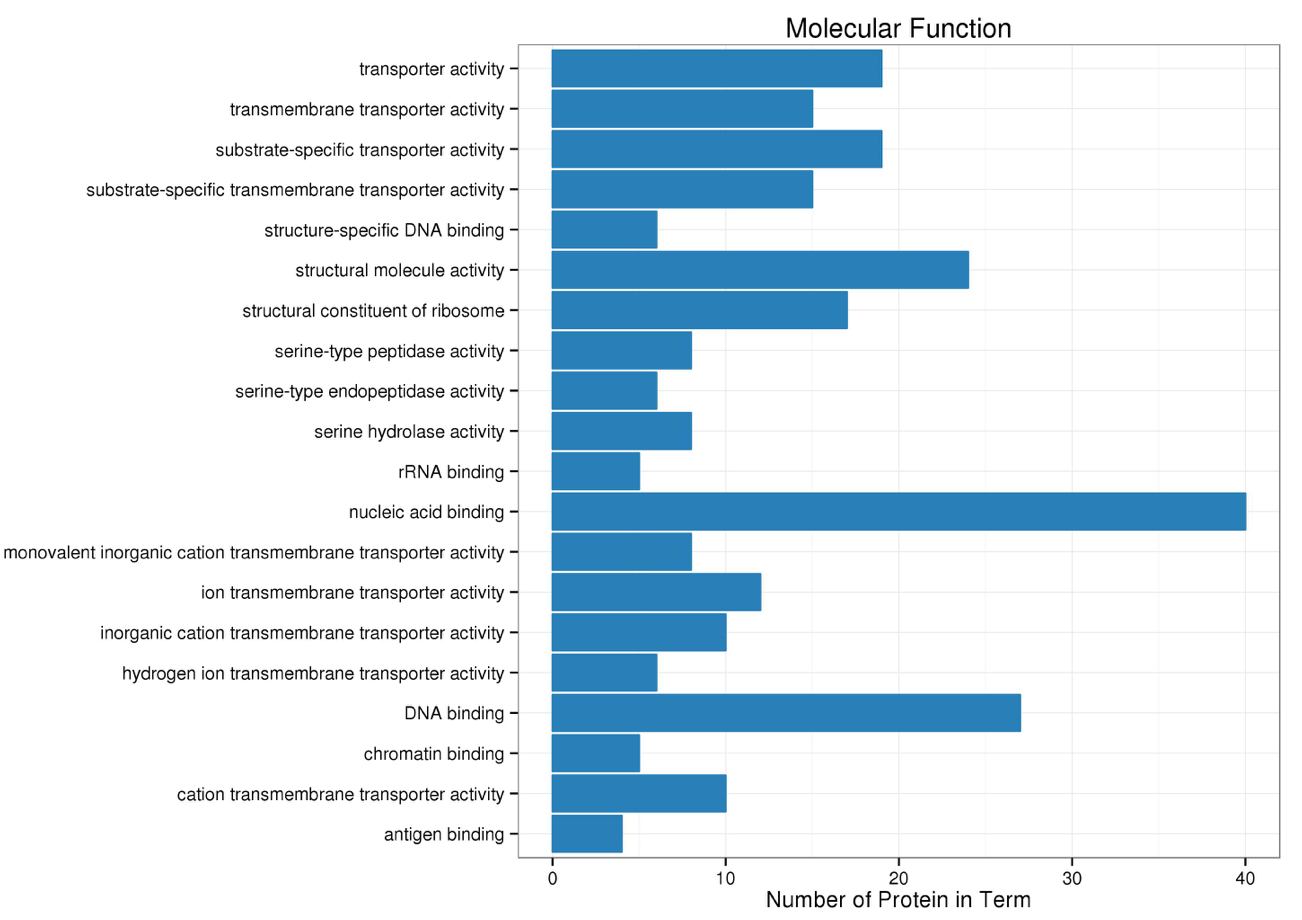


Figure S6


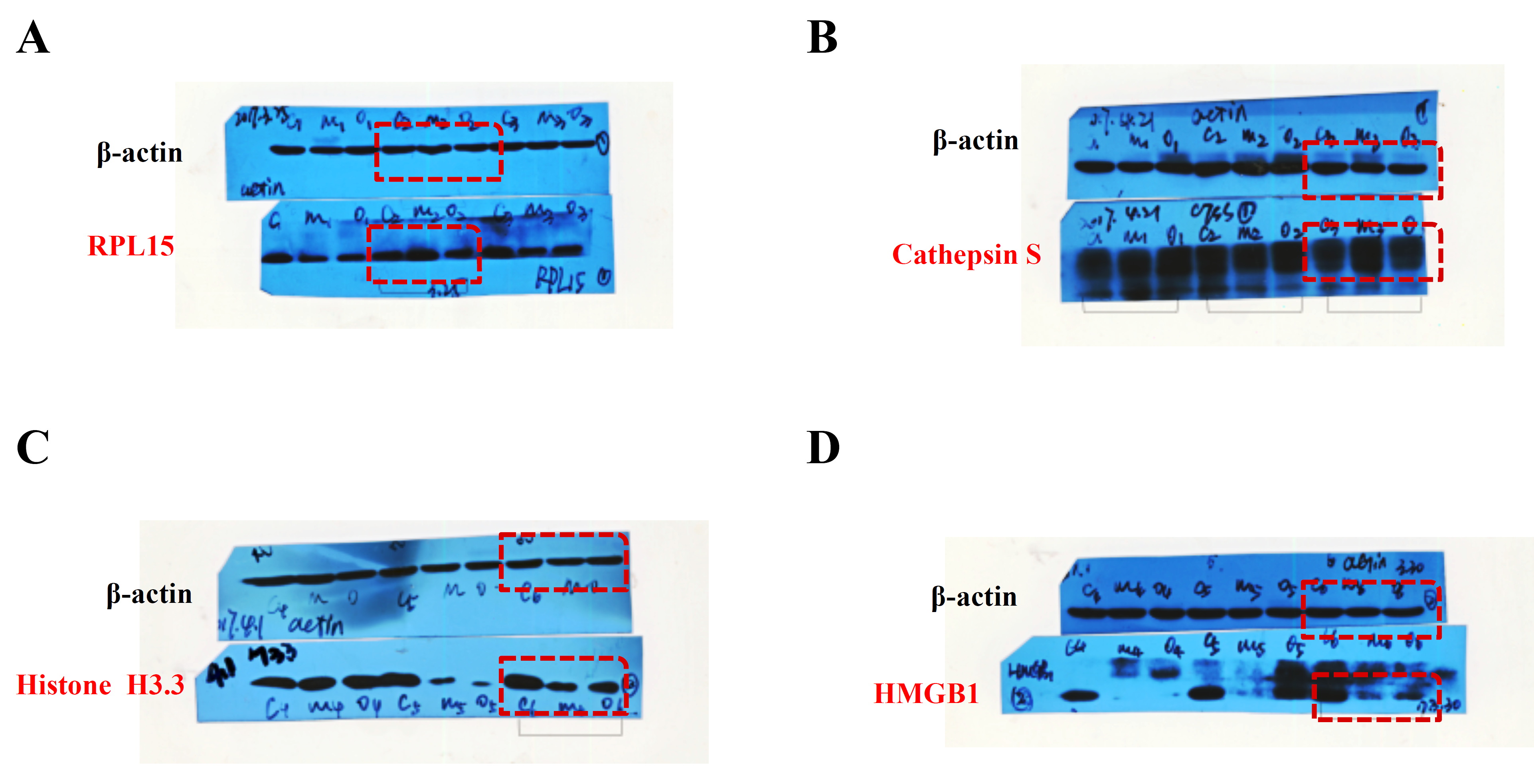


Figure S7
